# Security Considerations and Recommendations in Computer-Based Testing

**DOI:** 10.1155/2014/562787

**Published:** 2014-09-01

**Authors:** Saleh M. Al-Saleem, Hanif Ullah

**Affiliations:** ^1^Department of Information System, College of Computer and Information Sciences, King Saud University, P.O. Box 51178, Riyadh 11543, Saudi Arabia; ^2^Department of Computerized Based Testing, National Center for Assessment in Higher Education, P.O. Box 68566, Riyadh 11537, Saudi Arabia

## Abstract

Many organizations and institutions around the globe are moving or planning to move their paper-and-pencil based testing to computer-based testing (CBT). However, this conversion will not be the best option for all kinds of exams and it will require significant resources. These resources may include the preparation of item banks, methods for test delivery, procedures for test administration, and last but not least test security. Security aspects may include but are not limited to the identification and authentication of examinee, the risks that are associated with cheating on the exam, and the procedures related to test delivery to the examinee. This paper will mainly investigate the security considerations associated with CBT and will provide some recommendations for the security of these kinds of tests. We will also propose a palm-based biometric authentication system incorporated with basic authentication system (username/password) in order to check the identity and authenticity of the examinee.

## 1. Introduction

During the last few decades and especially from 1990 onwards computer-based testing (CBT) has become one of the most conspicuous ways of organizing and delivering the tests. The reason behind this prominence of CBT over the paper-and-pencil based testing is its ease of administration, immediate display of results, improved item development, enhanced identification and authentication, and so forth [1]. However, most of the organizations and institutions are still relying on the examinations where the examinees have face to face exams in an identified place under an administered situation. This may help the organizations to check the authenticity of the examinee by checking his identity using ID card and also ensuring that no cheating is going on during the exam [2]. But due to the enormous advantages of computer-based testing over the traditional paper-and-pencil based testing, most of the organizations are now moving towards CBT.

Due to the hasty and considerable development in the provision of detached and internet-delivered computer-based testing, a number of issues in the administration standards, security, and control over the testing processes are raised. The most important one is that of authentication of examinee and examiner. Both the examinee and examiner should be authenticated in order to secure the computer-based testing. Different techniques have been proposed for the authentication purpose. A theoretical approach was proposed by [3] that incorporates the fingerprint biometrics with e-learning environments in order to restrain the unethical conduct during the exams. The proposed solution would enhance the current authentication scheme by adding the fingerprint biometrics. The technique is randomly exercised during the exam with a very short fingerprint scanning response time in order to get additional security. The approach could discourage the learners and examinees from having someone else taking the exam for them.

Similarly a multimodal biometric approach was proposed by Asha and Cellappan in [4] in order to authenticate the examinees in the test. The proposed technique was a combination of fingerprint and mouse dynamics. The basic aim of the approach was to reduce the cheating during the exam.

Some other techniques have been used to authenticate the examiner or invigilator. The authenticity of the examiner is also more important because the examiner has access to the examinee registration data as well as the examinee test data. Different techniques have been proposed for the authenticity of the examiner. Moreover an e-monitoring based scheme has also been proposed to monitor the examinee during the exam and to rectify or detect the problem of cheating during the exam.

The remainder of this paper is organized as follows.  Section 2 describes the related work relevant to our paper.  Section 3 investigates privacy and security issues/considerations among the available computer-based testing standards.  Section 4 provides recommendations for secure computer-based testing environment and elaborates the proposed palm-based authentication technique.  Section 5 offers conclusions.

## 2. Related Work

Deepthi et al. proposed a novel approach to enhance the security for online exams by introducing the idea of group cryptography with an e-monitoring scheme. Through the e-monitoring system the examinees can be monitored similar to the offline exams by webcams [5]. They also proposed different methods to detect and prevent ongoing exam cheating like the identities of the entities on the system that are verified by webcams and these reference photos taken during the verification process are stored for further authentication during the exam. Similarly the examinees monitoring data are recorded and stored during the exam. Also screen shots are taken during the exam so that the proctor can better determine the status of the examinee during the exam [5].

Castella-Roca et al. proposed a secure e-exam management system where all data and information should be in digital format. Also they proposed a cryptographic scheme that should be executed at every stage of exam in order to get the maximum security [2]. Their system is based on different cryptographic protocols offering high level of security during the entire exam. The authors identified different stages of exam, for example, setting up an exam, beginning, holding and submitting, grading, obtaining, and revising the exams. They also identified different security requirements like authenticity, privacy, correction, secrecy, receipt, copy detection, and so forth [2]. The authors also proposed that the exam should be in a supervised environment.

Oluwatosin and Samson examined some of the challenges of the existing computer-based testing systems and came up with a new system that could be deployed on either internet or intranet. The proposed scheme was designed by unified process using UML [6]. Security and result integrity features were integrated in the system. The examiner should answer some questions on the first login and should update his authentication details. All authentications are logged automatically by the system in order to determine any illegal access. Also an examinee could not have more than one active session running simultaneously.

Anusha et al. studied various authentication techniques like unimodel, multimodel, and data visualization and proposed a method called enhanced security using data visualization (ESDW) for online exams [7]. The method included the examinee authentication at the beginning of the exam and continuous monitoring through a webcam during the entire exam. The authentication process of the examinee was first carried out by preprocessing of the image through filtering, normalization, and segmentation. The extraction of feature was done based on the color, texture, and shape of the desired image.

El-Khatib and Korba examined the provisions and limitations for privacy and security by investigating some of the most popular e-learning standards. The capabilities of many existing privacy enhancing technologies including network privacy, policy-based privacy, and trust systems were reviewed and assessed [8]. The problems of privacy and security for distributed e-learning systems, where the learner can access the learning contents from anywhere, using any suitable device, that is, desktop computers, PDA, and so forth, were also investigated. Overall privacy requirements for e-learning systems based on “privacy principals" were highlighted.

Huszti and Pethő described a cryptographic scheme that retains the security requirements without the intervention of a trusted third party. Authenticity, anonymity, secrecy, robustness, and correctness are the main security features highlighted in this paper. These requirements were accomplished by applying cryptographic primitives [9].

Similarly Chang and Ansley [10] investigated and compared the properties of item exposure control methods in order to estimate the abilities of examinees in computer adaptive testing context. Different item pool sizes and different desired maximum exposure rates, effectiveness, and psychometric properties of the various control methods were evaluated in terms of test security, item overlap rate, and so forth; the objective of the study was to offer more information about the properties of the various exposure control strategies, to provide information about how the exposure control methods would be affected by using different sizes of item pools, and to provide guidelines for selecting a specific exposure control method.

Asha and Chellappan proposed a multimodel biometric technique in [4] for the authentication of e-learner in an e-learning environment. They classified the authentication system into three categories, that is, method based on human memory, method based on physical devices, and method based on biometrics. The proposed approach incorporated the fingerprint biometrics with the behavioral biometrics or mouse dynamics. These mouse dynamics were collected passively and verified throughout the session. The authors concluded that the proposed approach would enhance the authentication process.

Levy and Ramim presented a theoretical approach for biometric authentication of e-exams in [3]. The proposed approach used a fingerprint biometric solution in e-exams. During the exam, the learner's access is authenticated once at login for the whole duration. The authors proposed that the current authentication system would be enhanced by using the fingerprint biometrics solution.

In [11–14] the authors proposed different palm-based authentication techniques. In [11] the authors proposed a scanner-based personal authentication system using the palmprint features. The proposed technique suits for many network based systems. Preprocessing, feature extraction, and modeling modules were used to generate the matching templates. Similarly in [12–14] the authors used wavelet-based, symbolic representation for palm-based authentication methods.

In paper [15–17] the authors proposed a genetic algorithm based palm recognition method for authentication process. The authors suggested that fingerprint and facial recognition systems are slow and required more expensive technical equipment, while palm recognition method did not require special equipment and could be used in systems where fast detection is required. Similarly a hand-based and palmprint and iris-based authentication system were proposed in [18–20]. Palmprint and iris-based authentication were used in order to overcome the problem of dictionary attacks.

## 3. Security Considerations

Many organizations and institutions around the world are moving towards computer-based testing from traditional paper-and-pencil based testing. However, this move will require substantial resources in terms of cost, management and administration, and security considerations. For a successful movement, it is recommended to prepare a plan and to highlight all of the above mentioned issues. In this paper we are going to highlight all the considerable issues related to the security of computer-based testing (CBT).

### 3.1. Required Resources for CBT

A number of resources are required for CBT administration. These resources include the cost associated with items such as computers, printers, and handheld devices although the costs related to traditional test or paper-and-pencil based tests are removed, but still the cost for CBT is much higher than the other. Moreover the cost associated with the online exam management systems and the licensing cost for different online/desktop item bank creation software's should also be considered in order to shift from paper-and-pencil based testing environment to CBT.

### 3.2. Item Bank Creation and Exposure Control

One of the main issues related to CBT is the creation and administration of item banks. CBT normally requires a huge number of item banks because of its frequent scheduling. Creation of item banks by subject-matter experts (SMEs) and their related security is one of the most important issues. The SMEs should be a trusted body and should not provide the detail about the item banks to a third party or to the examinee directly. The second main issue with item banks is its exposure control. The issue arises when some items appear more frequently within a short period of time or within a specific geographical area.

Some of the test-takers or examinees may have access to these frequent appearing test items before the test schedule and these test items could be compromised. This may result a significant concern for high stake test makers [10].

Several algorithms have been proposed in order to control item exposure rates. The algorithm in [21] employs an exposure control parameter for each item in the bank determined by a series of repetitive simulations. For items appearing rarely, the associated exposure control parameters could be as high as 1.0, meaning that once these items are selected, they are almost presented.

Similarly the algorithm proposed in [22, 23] for unconditional multinomial procedure was derived from the Stocking and Lewis approach. Rather than using the procedure of Stocking and Lewis, they used the idea of multinomial model for the selection of next item that has to be administered. They also presented an algorithm by using conditional multinomial procedure to control the item exposure to the examinees at similar level of proficiency [23].

### 3.3. Identification and Authentication of Examinee

Test security is one of the most important aspects of any exam, whether it is administered as paper-and-pencil based or CBT. The primary element of security in CBT is the procedure used to identify and authenticate the examinee. Different people used different ways to identify the examinee.

A range of levels of authentication could be used for the examinee [24]. The authentication could be exercised by username and password. Also the access could be limited to specific machines over the internet by allowing specific IP addresses. Both of these methods could also be merged in order to get high level of authenticity. Further enhancements could be made to the system by adding fingerprint and retinal eye-pattern recognition. Moreover high stake exams would require the presence of an administrator to check the identity of the examinees and to make sure that the exam is completed under good conditions.

A multimodal biometric approach was introduced in [4] for the authentication process of examinees. They used the combination of fingerprint and mouse dynamics. Mouse with a fingerprint scanner could be used in order to capture the fingerprint of the examinee along with the measurement of mouse dynamics. The examinee would be authenticated once by the server at login for the whole duration of the activity session. The proposed solution would enhance the authentication process and would reduce the chances of cheating during the exam.

Another biometric recognition system was applied to evaluate the basic knowledge in high school students in [25]. The assessment was carried out in order to answer the main problem, who is there? The authentication process was assessed by means of index fingerprint. During the enrollment process the proposed scheme saved the student fingerprint and indexed it in the features database. Student personnel ID was assigned in the features database in order to link the students personnel information with the fingerprint image.

A theoretical fingerprint biometric authentication system was presented in [3] for the delivery of e-learning courses. In their approach they incorporated the existing fingerprint biometric authentication technologies with e-learning environments to restrain unethical behavior during exam taking. The approach suggested practical solutions to incorporate a random fingerprint biometrics user authentication during exams. The solution will enhance the current authentication system by adding the fingerprint biometric solution.

### 3.4. Invigilator/Proctor Authentication

Besides the examinee authentication, invigilator or proctor authentication is also an important aspect in online or computer-based tests, because the proctor has access to many aspects of the exam, including the examinee registration data, test data, or examinee test. In both of these cases either the examinee data or the examinee test data could be compromised. In order to rectify this problem, an invigilator code should be generated along with the examinee code, so that the invigilator would never have access to the examinee code and, therefore, have no access to the examinee test. Also the invigilator could be authenticated by providing secure connection to the invigilator computer during username/password authentication. A role-based access control could also be used in order to keep the access secure [26].

### 3.5. Cheating during Exam

Another important security issue in the computer-based testing is cheating during the ongoing exam. The examinee can cheat either by communicating with their other colleagues or by browsing over the internet. To overcome this problem, a continuous monitoring system should be implemented in order to monitor the examinee throughout the exam. Different people have used different techniques for the same problem. In [7] the authors proposed the use of webcams to monitor the examinees during the exam. Also this monitoring data should be stored on a monitor server for future use. The authors continuously monitor and save the recording of the entire exam in order to rectify the problem of cheating. Also screen shots of the examinee computer were also saved in parallel with video in order to better determine what actually the examinee was doing with his computer.

Another way to rectify this problem is to arrange the test in a web lock environment, where the test is displayed in a full screen option and the browser and other applications are locked during the exam. The examinee can only minimize or exit the full screen once the test is submitted for grading. Also print, print screen and capturing options, copy and paste, right-click menu options, browser menu and toolbar options, function keys, and so forth are all disabled during the exam.

### 3.6. E-Monitoring System

E-Monitoring system is a system where the examinee or the student is monitored by a webcam during the entire exam. This system is one of the best solutions to rectify the problem of cheating during the exam. The system could be implemented and centralized, where the examinees of different centers are monitored through a centralized system from a particular place, or decentralized where each and every test center has its own monitoring system inside the test center and the examinees are monitored from there. This video streaming of the entire exam is stored on different hard drives for future use.

## 4. Proposed Authentication Scheme

**Figure 1:**
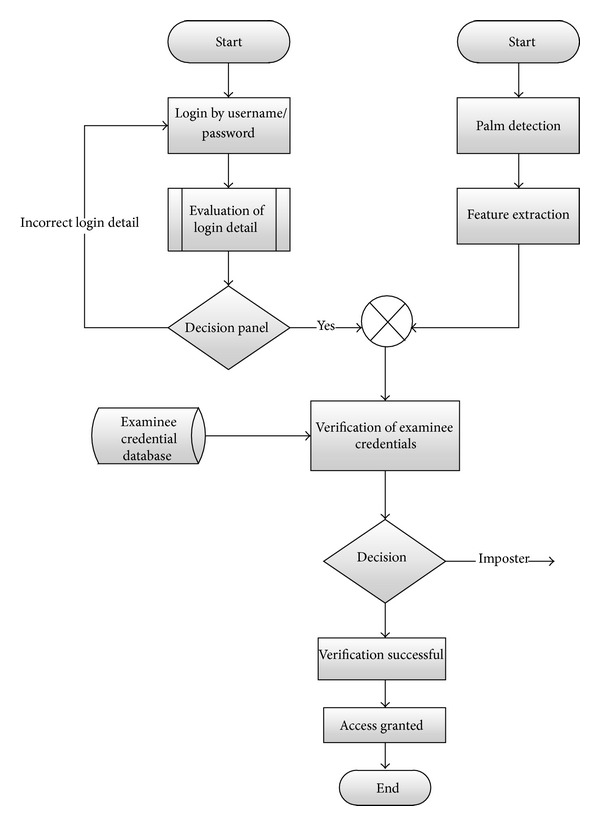
Proposed biometric authentication scheme.

It is necessary to combine many biometric traits to secure computer-based tests. Many models and schemes have been discussed in the previous section for the same purpose. In this section we are going to propose a new authentication scheme for computer-based tests.  Figure 1 summarizes the entire biometric authentication scheme. The proposed scheme incorporates the traditional username/password technique to the palm-based biometric authentication technique in order to get highest level of security during the exams. The examinee will first enter his/her login detail. These credentials will be evaluated and if it is correct, the examinee will be asked to put his palm on the device for the second phase of the authentication process; otherwise, a message will be displayed with incorrect username/password. After the palm detection process, some of the features of palm will be extracted in order to check it against the features in the database.

Once the features are extracted, they are checked for the verification of examinee. If the verification is successful, the examinee will be permitted to the exam; otherwise, the examinee will be instructed that he/she is not an authorized examinee. Also in order to get the optimum security during the exam, the examinee will be continuously monitored by a webcam to avoid any inconvenience during the exam. The webcam monitoring will detect the threat of cheating during the exam. Also the authentication process can be enhanced by the monitoring scheme. The username and password are basically an exam specific generated codes or credentials that are produced during the test creation process. For each and every examinee a separate exam ID or username and password are generated. When the examinee arrives at the specified center, these credentials are provided to the examinees. Also the basic identification process (checking of ID cards or other identification documents) is carried out at the reception of the exam center. Once all of these requirements are fulfilled, then the examinee is asked to put his palm on the device for the second phase of the authentication process.

For the entire process secure socket layer (SSL) will be used because the secure tunnels that can be created with SSL offer a means to exchange the authentication information or credentials without it being easily intercepted. The proposed method will handle the basic authentication problems like someone else is trying to attempt the exam instead of actual examinee.

The proposed technique is different from all other techniques because of its emergence. Palm-based authentication is latest trend instead of fingerprint and other authentication methods. Also the proposed technique will be implemented in our center in the near future and we have already the devices for palm-based authentication system. Moreover the retinal authentication is an expensive and difficult solution because the devices are costly and not easily available in the market. Also the entire authentication and verification process for the retinal system is much difficult than palm-based authentication and verification method.

## 5. Results and Discussion

**Figure 2:**
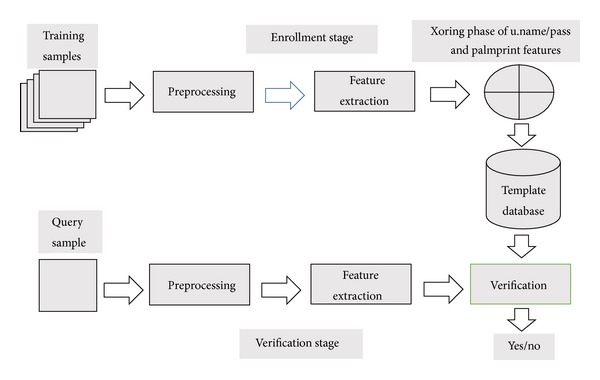
Enrollment and verification stage of the proposed system.

**Figure 3:**
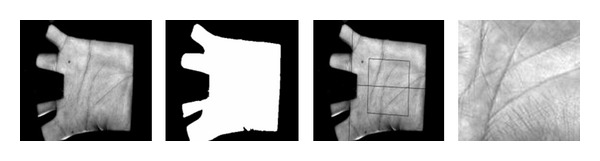
Images from the IIT Delhi database.

The results of the proposed palm-based authentication scheme are described in this section. The block diagram of the enrollment and verification stage is also given in Figure 2. In the preprocessing stage of the enrollment and verification stage of the palm, region of interest (ROI) of the image is obtained. The images are taken from the IIT Delhi palmprint database and are given in Figure 3. After the preprocessing stage, the desired features of the given images are extracted and are stored in a database in order to XOR (XOR or Exclusive-OR is the process of combining the bits extracted from region of interest of the palm image and the bits obtained from the username and password in order to make the process secure and strong) with the username and password of the selected examinee. All these credentials are then stored in a database for the verification process. The same process is repeated for the verification process. Once the features are extracted, they are verified with the stored features in the database; if both the features are matched, the verification is successful; otherwise the verification process is denied.

The proposed approach is tested with different sets of input images and there results are stored in the template database for further processing. The detailed results and further discussion of the proposed scheme will be included in the future work and will be a part of the next paper on the same topic.

## 6. Conclusion and Future Work

In this paper we have explored many security considerations in computer-based and online-based testing. Also we have explored many authentication schemes for the authentication of examinees. Moreover we have proposed a new authentication scheme that incorporates the username/password with the palm-based biometrics. The proposed scheme will enhance the authentication process up to an optimum level during the computer-based or online-based testing. The video capturing technique will also be merged with the existing system, in order to make it more secure in the future. Also CATSIM simulation tool will be used to simulate the entire authentication and verification process.
